# Frosted branch angiitis as a result of immune recovery uveitis in a patient with cytomegalovirus retinitis

**DOI:** 10.1186/1869-5760-3-52

**Published:** 2013-06-22

**Authors:** Supinda Leeamornsiri, Pitipol Choopong, Nattaporn Tesavibul

**Affiliations:** 1 Department of Ophthalmology, Thammasat University Hospital, Pathumthani, Thailand; 2 Department of Ophthalmology, Siriraj Hospital, Mahidol University, Bangkok, Thailand

**Keywords:** Frosted branch angiitis, Immune recovery uveitis, Immune recovery vitritis, Cytomegalovirus retinitis

## Abstract

**Background:**

Since the introduction of Highly Active Antiretroviral Therapy (HAART), AIDs related morbidity and mortality have declined. However, the advent of HAART brought the new problem of immune recovery inflammatory syndrome. Cytomegalovirus retinitis remains the most common cause of visual loss in AIDs patients. Some patients with cytomegalovirus retinitis who experienced immune recovery as a consequence of HAART develop worsening of visual symptoms from immune recovery uveitis (IRU).

**Findings:**

We report a case of cytomegalovirus retinitis and AIDs who developed an unusual presentation of IRU after the initiation of HAART. A 40-year-old woman presented with a history of blurry vision in the right eye. She was diagnosed with human immunodeficiency virus infection and cytomegalovirus retinitis, treated with intravitreal injections of ganciclovir. The retinitis improved. One week after HAART initiation, she developed IRU, characterized by increased intraocular inflammation, extensive frosted branch angiitis and cystoid macular edema. The CD4+ T lymphocyte count increased from 53 to 107 cells/mm^3^. Systemic prednisolone with continuation of HAART and intravitreal injections of ganciclovir were given with significant improvement.

**Conclusion:**

Atypical presentation of IRU, characterized by extensive frosted branch angiitis and increased intraocular inflammation may occur in immunocompromised patients with cytomegalovirus retinitis who experienced immune recovery. The time from HAART initiation to develop IRU may vary from days to months. This case demonstrated a very rapidly developed IRU which should be recognized and appropriately managed to avoid permanent damage of the eye.

## Findings

### Introduction

Since the introduction of Highly Active Antiretroviral Therapy (HAART), AIDs related morbidity and mortality have declined. This therapy accounts for the recovery of the immune system, manifested by an increase in the number of the CD4+ T lymphocyte counts and a decrease in human immunodeficiency virus (HIV) viral loads. However, the advent of HAART brought the new problem of immune recovery inflammatory syndrome (IRIS), characterized by paradoxical worsening of treated opportunistic infection or unmasking of subclinical, untreated infection [1,2]. Ocular IRIS is referred to as immune recovery uveitis (IRU) [3].

Cytomegalovirus retinitis remains the most common cause of visual loss in AIDs patients either pre or post HAART era [4,5]. Some patients with cytomegalovirus retinitis who experienced immune recovery as a consequence of HAART develop worsening of visual symptoms from IRU. The pathogenesis of IRU remains to be elucidated. However, it has been postulated that IRU may represent the exaggerated and dysregulated cellular immune response to cytomegalovirus antigens in the eye by HAART mediated improvement of immune function [2,6,7]. We report here a case of cytomegalovirus retinitis and AIDs who developed an unusual presentation of IRU after the initiation of HAART.

### Case report

**Figure 1:**
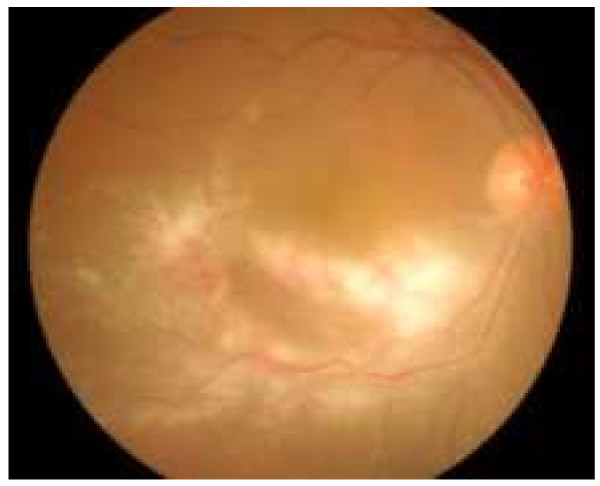
**Cytomegalovirus retinitis (CMVR).** Patient presented with multiple large areas of retinitis and intraretinal hemorrhage involving the inferotemporal retina.

**Figure 2:**
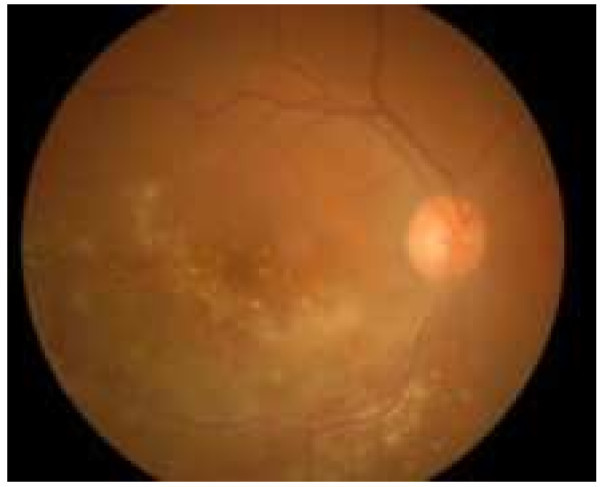
**At a 6-week follow-up, the retinitis had improved.**

**Figure 3:**
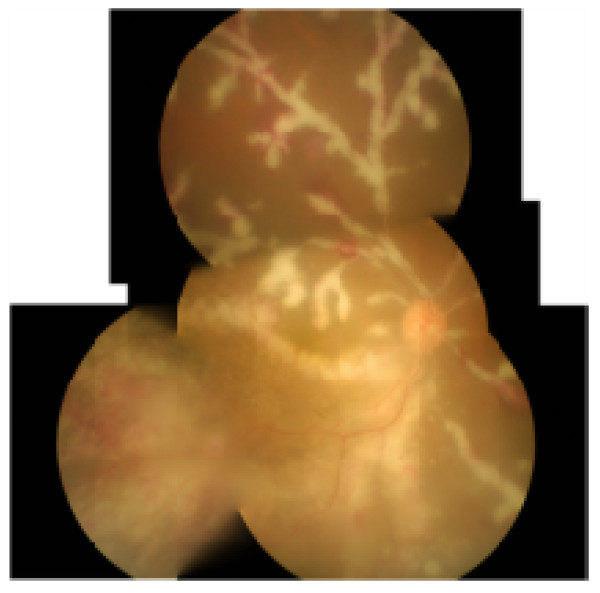
**After 1 week of HAART initiation (a 7-week follow-up).** The patient developed immune recovery uveitis, characterized by increased intraocular inflammation, extensive frosted branch angiitis, and cystoid macular edema.

**Figure 4:**
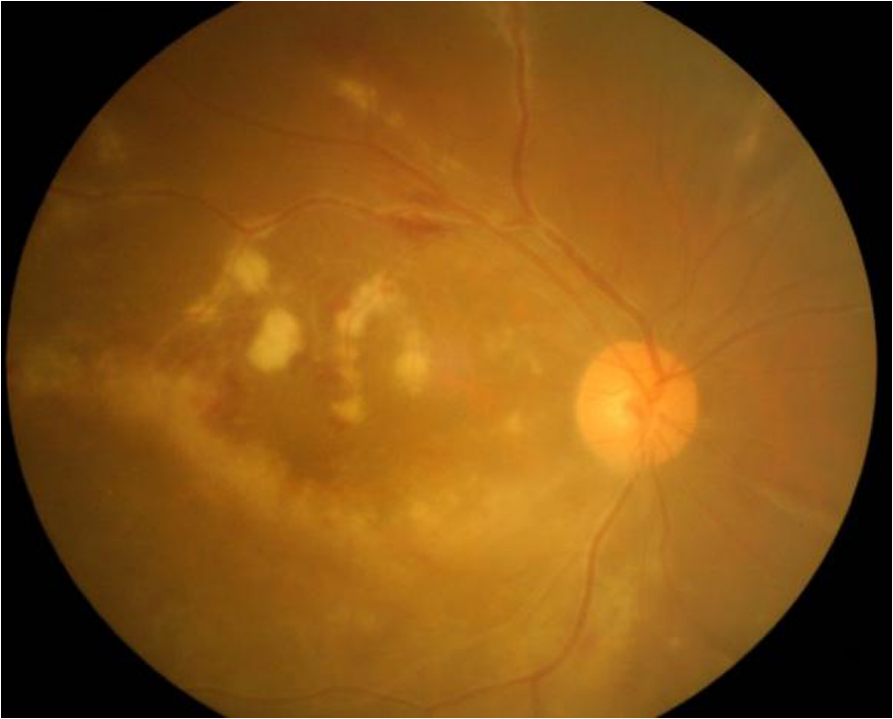
Frosted branch angiitis had significantly improved after 1-week prednisolone therapy.

**Figure 5:**
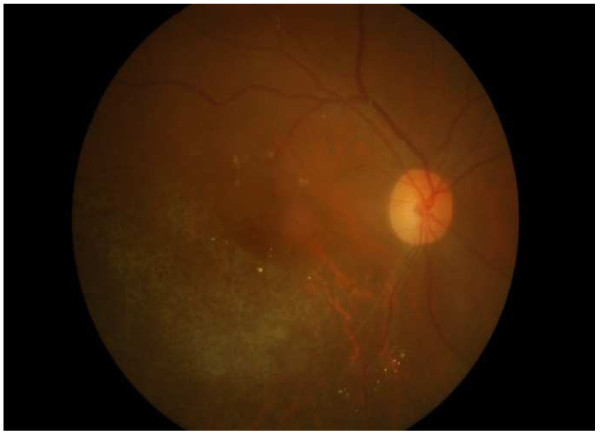
**At a 5-month follow-up, the retinitis had resolved.** Ganciclovir therapy was discontinued.

A 40-year-old woman presented in July 2010 with a complaint of blurry vision in the right eye. Right ocular examination disclosed a visual acuity of 20/100. The anterior segment was unremarkable. Multiple large areas of retinitis with intraretinal hemorrhage involving the inferotemporal retina were noted. Cytomegalovirus retinitis was clinically diagnosed (Figure 1). The left eye was unremarkable. The anti-HIV test was positive and the initial CD4+ T lymphocyte count was 53 cells/mm^3^. Systemic anti-cytomegalovirus medication was limited because of financial issue. Therefore, a weekly intravitreal injection of 2 mg/0.04 ml ganciclovir was given. The retinitis had improved with a visual acuity of 20/40 at a 6-week follow-up (Figure 2). At patient’s 7-week follow-up, the initiation of highly active antiretroviral therapy (HAART; Nevirapine, Lamivudine, and Stavudine) was given. One week later, her right visual acuity decreased to 5/200. Right ocular examination revealed 1+ aqueous cells and 1+ vitreous haze. Extensive frosted branch angiitis and cystoid macular edema were noted. The inferior retina was swollen (Figure 3). OCT revealed marked fluid accumulation in the macular area. The left eye was unremarkable. At this time, her CD4+ T lymphocyte count was 107 cells/mm^3^. She was diagnosed with immune recovery uveitis (IRU). Treatment commenced with intake of 25mg/day oral prednisolone (0.5 MKD) and continuation of HAART and intravitreal ganciclovir injections lead to significant improvement of perivascular infiltration within 1 week (Figure 4). The macular edema gradually improved. At a 5-month follow-up, the area of retinitis had been resolved and the CD4+ T lymphocyte count was 169 cells/mm^3^. Intravitreal ganciclovir therapy was discontinued (Figure 5). Her vision became 20/40 at a 1-year follow-up. The CD4+ T lymphocyte count was 213 cells/mm^3^.

### Discussion

Among the era of HAART, IRU has become the cause of visual loss in patients with cytomegalovirus retinitis. Kempen et al. reported a prevalence of IRU approximately 20% among those who had developed immune reconstitution [8]. Currently, there have been no definite criteria of IRU. It has been generally recognized by new or increased intraocular inflammatory reaction in patients with AIDS and cytomegalovirus retinitis receiving HAART. The inflammation was associated with an increase in the CD4+ T lymphocyte counts of at least 50 cells/mm^3^ to the level of 100 cells/mm^3^ or more [3]. The clinical presentation of IRU may vary from mild to severe vitritis, macular edema, epiretinal membrane formation, posterior synechiae, neovascularization of the retina or optic disc, and papillitis [9-11]. Although in HIV-infected patients, frosted branch angiitis is commonly associated with cytomegalovirus infection and the administration of anti-cytomegalovirus therapy without the need for corticosteroids results in the resolution of the angiitis [12,13]. Our case demonstrated frosted branch angiitis as a sign of IRU as it occurred after the patient reached immune recovery state and significantly improved after systemic corticosteroid therapy. Since the patient developed IRU while cytomegalovirus was not completely resolved, as shown by the area of active retinitis, we decided to continue anti-cytomegalovirus medication concomitant with prednisolone therapy. A previous report by Alp et al. also described a HIV-infected patient with cytomegalovirus retinitis who developed intraocular inflammation, frosted branch angiitis and some areas of retinitis after HAART initiation. However, the patient was diagnosed with IRU despite the CD4+ T lymphocyte count less than 50 cells/mm^3^[14].

The median time from HAART initiation to develop IRU has varied from 20 to 43 weeks [6,15]. Our patient had IRU in only 1 week after HAART initiation. To date, this case has reported the earliest onset of IRU after HAART initiation.

The low CD4+ T cell count at the initiation of HAART and the large area of cytomegalovirus retinitis were associated with an increased incidence of IRU [2]. With this patient, the CD4+ T lymphocyte count doubled at 1-week HAART therapy, and then, the rate of increasing CD4+ T lymphocyte count declined (53, 107, 169, and 213 cells/mm^3^ at 1 day, 1 week, 5 months, and 1 year, respectively). It was possible that a rapid increase in the number of these dysregulated CD4+ T lymphocytes as a consequence of HAART may be another risk factor of IRU. The rapid rising of CD4+ T lymphocyte count at 1 week of HAART therapy may be attributable to redistribution of lymphocytes from lymph nodes into blood. In HAART naïve HIV infection, proinflammatory cytokines and adhesion molecules increase in lymph nodes that result in trapping of lymphocytes in lymphatic tissue [16,17]. After HAART initiation, proinflammatory cytokines and adhesion molecules decrease, in parallel with an initial increase in peripheral CD4+ lymphocytes [16-18].

### Conclusions

Atypical presentation of IRU, characterized by extensive frosted branch angiitis and increased intraocular inflammation may occur in immunocompromised patients with cytomegalovirus retinitis who experienced immune recovery. The time from HAART initiation to develop IRU may vary from 1 week which is the earliest onset in this case to several months. Prompted diagnosis and appropriate treatment should be warranted for preventing permanent visual damage.

### Consent

Written informed consent was obtained from the patient for publication of this report and any accompanying images.

## Abbreviations

HAART: Highly active antiretroviral therapy; IRIS: Immune recovery inflammatory syndrome; IRU: Immune recovery uveitis.

## Competing interests

The authors declare that they have no competing interests.

## Authors’ contributions

SL, PC, and NT participated in the drafting of the manuscript. SL participated in the management of the patient. All authors read and approved the final manuscript.
